# Homocysteine-induced peripheral microcirculation dysfunction in zebrafish and its attenuation by L-arginine

**DOI:** 10.18632/oncotarget.16811

**Published:** 2017-04-04

**Authors:** Sang Joon Lee, Sung Ho Park, Jinhyuk Fred Chung, Woorak Choi, Hyung Kyu Huh

**Affiliations:** ^1^ Center for Biofluid and Biomimic Research, Department of Mechanical Engineering, Pohang University of Science and Technology (POSTECH), Pohang, South Korea; ^2^ Xylonix Pte Ltd, Singapore, Singapore

**Keywords:** homocysteine, L-arginine, peripheral microcirculation dysfunction, inflammation, zebrafish

## Abstract

Elevated blood homocysteine (Hcy) level is frequently observed in aged individuals and those with age-related vascular diseases. However, its effect on peripheral microcirculation is still not fully understood. Using *in vivo* zebrafish model, the degree of Hcy-induced peripheral microcirculation dysfunction is assessed in this study with a proposed dimensionless velocity parameter ${\bar{\text{V}}}_{\text{CV}}/\;{\bar{\text{V}}}_{\text{PCV}}$, where ${\bar{\text{V}}}_{\text{CV}}$ and ${\bar{\text{V}}}_{\text{PCV}}$ represent the peripheral microcirculation perfusion and the systemic perfusion levels, respectively. The ratio of the peripheral microcirculation perfusion to the systemic perfusion is largely decreased due to peripheral accumulation of neutrophils, while the systemic perfusion is relatively preserved by increased blood supply from subintestinal vein. Pretreatment with L-arginine attenuates the effects of Hcy on peripheral microcirculation and reduces the peripheral accumulation of neutrophils. Given its convenience, high reproducibility of the observation site, non-invasiveness, and the ease of drug treatment, the present zebrafish model with the proposed parameters will be used as a useful drug screening platform for investigating the pathophysiology of Hcy-induced microvascular diseases.

## INTRODUCTION

Homocysteine (Hcy) is a sulfur-containing amino acid and a byproduct of methionine metabolism. Elevated blood Hcy level (hyperhomocysteinemia) is frequently observed in aged individuals and those with age-related vascular diseases, including cardiovascular and cerebrovascular diseases [1]. A proposed mode of Hcy-induced vascular disease is to impair the endothelial synthesis of nitric oxide (NO) with subsequent onset of endothelial dysfunction that causes adherence of leukocytes to endothelial cells, activation of platelets, and inhibition of endothelium-mediated vasodilation [2–4].

In previous *in vivo* studies using mammalian animal models of rats and cynomolgus monkeys, diet-induced hyperhomocysteinemia was reported to reproduce the proposed Hcy-induced endothelial injuries and develop microvascular resistance from increased endothelial adherence by neutrophils [2, 5]. Specifically, the endothelial adherence by neutrophils upon hyperhomocysteinemia is accelerated by upregulating the P-selectin, L-selectin, intercellular adhesion molecule-1 (ICAM-1), and CD11b/CD18 [6, 7]. These results support the observed endothelial injuries and peripheral microcirculation dysfunction (PMD) development by the increased microvascular accumulation of neutrophils [8]. Unfortunately, the extent of PMD arising from such changes is only qualitatively investigated, because mammalian animal models require invasive measurement techniques and post-mortem examinations [9]. Therefore, the development of a viable *in vivo* animal model that provides quantitive assessment of PMD would open new opportunities in the studies on microvascular diseases and corresponding drug screening.

Zebrafish *(Danio rerio*) as a vertebrate is an emerging disease model used for understanding human genetics and embryonic development due to its high similarity with human genome [10, 11]. Given its optical transparency and hemodynamic analogy with human capillary networks, it has been also used as a non-invasive model for real-time *in vivo* investigation of cardiovascular morphologies and pathologies, including the angiogenic effects of elevated Hcy [12, 13].

In the present study, hemodynamic effects of Hcy treatment on microcirculation in zebrafish are experimentally investigated by a micro-particle imaging velocimetry (PIV) technique and digital motion analysis. The proposed dimensionless parameter ${\bar{\text{V}}}_{\text{CV}}/{\bar{\text{V}}}_{\text{PCV}}$ is used as a quantitative measure of the PMD severity. In addition, the protective effect of L-arginine against Hcy-induced PMD is also examined in zebrafish models with the proposed parameter.

## RESULTS

### Selection of vessels and definition of velocity parameters

**Figure 1:**
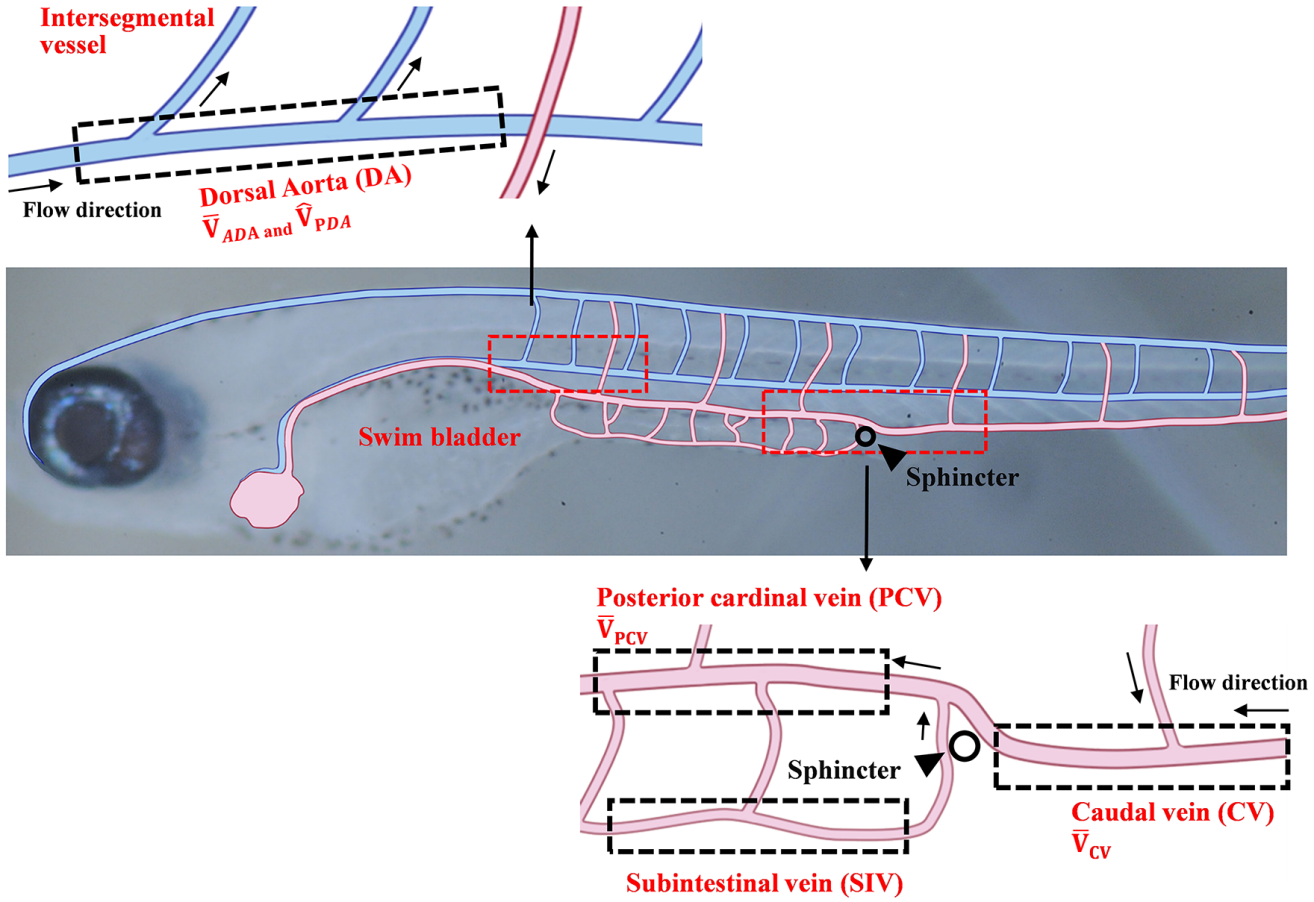
Schematics of the vascular network of a 10 dpf zebrafish Vessels are composed of the dorsal artery (DA), posterior cardinal vein (PCV), caudal vein (CV), and subintestinal vein (SIV). Each region of interest (ROI) is represented by dotted box. The time-averaged RBC velocity and peak systolic RBC velocity in the DA are defined as ${\bar{\text{V}}}_{\text{ADA}}$ and ${\hat{\text{V}}}_{\text{PDA}}$, respectively. The time-averaged RBC velocities in the PCV and CV regions are defined as ${\bar{\text{V}}}_{\text{PCV}}$ and ${\bar{\text{V}}}_{\text{CV}}$, respectively.

Schematic diagrams of the zebrafish vessel network are shown in Figure 1. A region of interest (ROI) in the dorsal aorta (DA) is designated behind the swim bladder from the first to the third segment of the bone. The time-averaged RBC velocity (${\bar{\text{V}}}_{\text{ADA}}$) and peak systolic RBC velocity (${\hat{\text{V}}}_{\text{PDA}}$) in the ROI of the DA are obtained by conditional phase averaging of data for 10 cardiac cycles. Veins are divided into the following three branches based on the location of the sphincter (black triangle): posterior cardinal vein (PCV), caudal vein (CV), and subintestinal vein (SIV). Similarly, the ROI in the PCV is designated at the left side of the sphincter where the bloods flowed from the CV and the SIV converge. Another ROI in the CV is designated at the right side of the sphincter. The time-averaged velocity of RBCs in the PCV and CV for 10 cardiac cycles is defined as ${\bar{\text{V}}}_{\text{PCV}}$ and ${\bar{\text{V}}}_{\text{CV}}$, respectively. The ROI in the SIV is designated under the PCV. These ROIs are marked by dotted boxes in the vessel diagram shown in Figure 1.

### Hcy-induced PMD in zebrafish at 10 days post fertilization (dpf)

The degree of Hcy-induced PMD after 24 h of Hcy exposure is assessed by dimensionless parameters: ${\bar{\text{V}}}_{\text{CV}}/{\bar{\text{V}}}_{\text{PCV}}$, ${\bar{\text{V}}}_{\text{CV}}/{\bar{\text{V}}}_{\text{ADA}}$, ${\bar{\text{V}}}_{\text{PCV}}/{\bar{\text{V}}}_{\text{PDA}}$, and ${\bar{\text{V}}}_{\text{PCV}}/{\hat{\text{V}}}_{\text{PDA}}$. The biological significance of the four non-dimensional velocities of zebrafish microcirculation is summarized in Table 1.

**Table 1:** Biological significance of dimensionless velocities

Dimensionless velocity	Biological meaning
${\bar{\text{V}}}_{\text{CV}}/{\bar{\text{V}}}_{\text{PDA}}$	Relative peripheral microcirculation perfusion level versus systemic perfusion
${\bar{\text{V}}}_{\text{CV}}/{\bar{\text{V}}}_{\text{PDA}}$	Relative peripheral microcirculation perfusion level versus average cardiac output
${\bar{\text{V}}}_{\text{PCV}}/{\bar{\text{V}}}_{\text{ADA}}$	Relative systemic perfusion level versus average cardiac output
${\bar{\text{V}}}_{\text{PCV}}/{\hat{\text{V}}}_{\text{PDA}}$	Relative systemic perfusion level versus peak cardiac output

**Figure 2:**
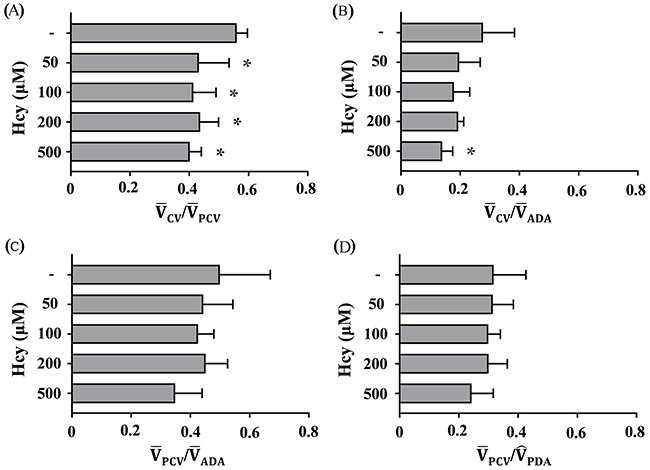
Hcy-induced PMD reduces blood flow in the CV The minimum concentration of 50 μM Hcy greatly reduces **(A)** ${\bar{\text{V}}}_{\text{CV}}/{\bar{\text{V}}}_{\text{PCV}}$. Meanwhile, the highest concentration of 500 μM Hcy significantly reduces **(B)** ${\bar{\text{V}}}_{\text{CV}}/{\bar{\text{V}}}_{\text{ADA}}$. However, **(C)** ${\bar{\text{V}}}_{\text{PCV}}/{\bar{\text{V}}}_{\text{PDA}}$ and **(D)** ${\bar{\text{V}}}_{\text{PCV}}/{\hat{\text{V}}}_{\text{PDA}}$ do not noticeably change, indicating that systemic perfusion is relatively preserved. Asterisk (*) denotes significant decrease in peripheral microcirculation perfusion according to the level of Hcy-induced PMD, compared with the control. n=4-6 per Hcy group; * p<0.05 (one-way ANOVA with Newman-Keuls's post-test). Resulting values are expressed as mean ± SD (standard deviation).

Exposing zebrafish to the near-physiological concentration of 50 μM Hcy significantly reduces ${\bar{\text{V}}}_{\text{CV}}/{\bar{\text{V}}}_{\text{PCV}}$ from 0.560 to 0.432 (22.9%, p<0.05) (Figure 2A). This result is corroborated by significant reduction in ${\bar{\text{V}}}_{\text{CV}}/{\bar{\text{V}}}_{\text{ADA}}$ from 0.277 to 0.139 (49.8%, p<0.05) at the highest concentration of 500 μM Hcy (Figure 2B). On the contrary, the dimensionless parameters representing systemic perfusion level show a little or no changes. ${\bar{\text{V}}}_{\text{PCV}}/{\bar{\text{V}}}_{\text{PDA}}$ is decreased from 0.500 to 0.349 (30.2%, p>0.05) at the highest concentration (Figure 2C). In addition, ${\bar{\text{V}}}_{\text{PCV}}/{\hat{\text{V}}}_{\text{PDA}}$ exhibits no change under the same conditions, except at the highest concentration (23.3%, p>0.05) (Figure 2D). These results suggest that Hcy treatment for 24 hr selectively reduces the level of peripheral microcirculation perfusion, and hence indicate the emergence of PMD. Meanwhile, the assessment of Reynolds (Re=VDv) and Womersley numbers ($\text{Wo }=\;\frac{D}{2}{\left(\frac{\omega }{v}\right)}^{\frac{1}{2}}$) at DA does not show significant changes over all Hcy doses tested in this study. They remain at comparable levels to those observed in human microcirculation. This implies that the rheological and blood flow characteristics are not significantly changed under the conditions in this study (Table 2).

**Table 2:** Variations in non-dimensional parameters according to Hcy level

	Control (n=4)	50μM Hcy (n=6)	100μM Hcy (n=4)	200μM Hcy (n=4)	500μM Hcy (n=5)
**Reynolds number**	0.0038±0.0012	0.0037±0.0014	0.0037±0.0007	0.0036±0.0006	0.0038±0.0012
**Womersley number**	0.011±0.0018	0.012±0.0021	0.012±0.0007	0.013±0.0024	0.013±0.0007

### Increase of number ratio of RBCs in the SIV

**Figure 3:**
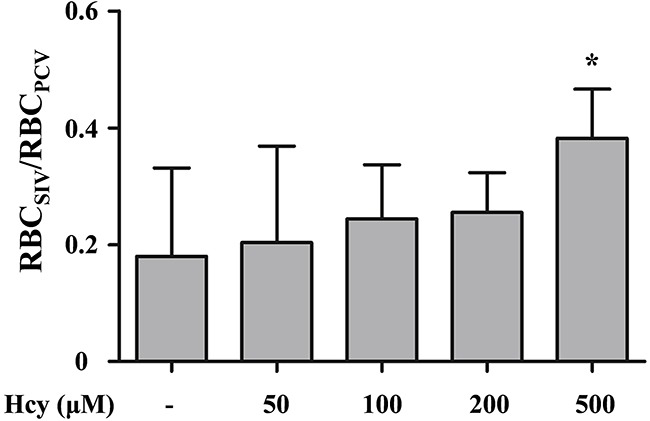
Variation in the number ratio of RBCs in the SIV and PCV regions The number ratio of RBCs passing through the SIV (RBC_SIV_) relative to the PCV (RBC_PCV_) is increased with the elevation of Hcy level for maintaining systemic perfusion against Hcy-induced PMD in the tail region. Asterisk (*) denotes significant increase in compensatory blood flow from the SIV. n = 4-6 per Hcy group; * p<0.05 (one-way ANOVA with Newman-Keuls's post-test). Resulting values are expressed as mean ± SD (standard deviation).

PMD occurrence is expected to increase blood flow in SIV to balance the reduced peripheral perfusion by virtues of the network Fahraeus effect and conservation requirement of RBCs in the closed circulatory system of zebrafish. This projection is checked by the changes in a dimensionless ratio, RBC_SIV_/RBC_PCV_, which is the ratio of RBCs passing through the center regions of the SIV to those of the PCV. The number of RBCs in each vein is evaluated by digital motion analysis technique [14]. RBC_SIV_/RBC_PCV_ is significantly increased as a result of Hcy-induced PMD in the tail region according to Hcy level from 0.180 upto 0.382 at the highest Hcy dose (500 μM) (p<0.05) (Figure 3).

### Pretreatment with 50 μM L-arginine prevents Hcy-induced PMD

**Figure 4:**
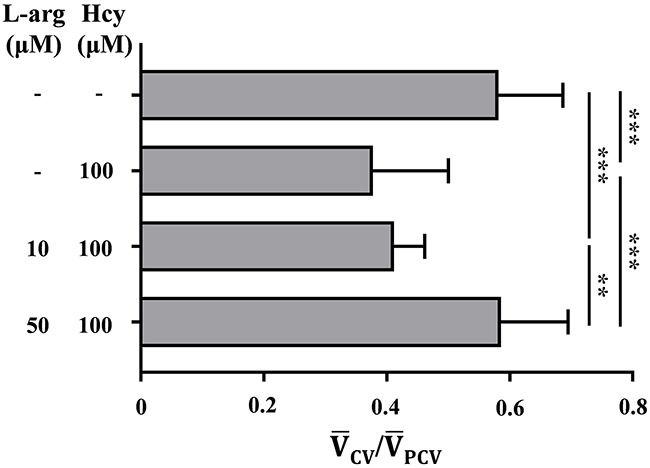
Pretreatment with 50 μM L-arginine attenuates the onset of Hcy-induced PMD The degree of PMD is assessed with the non-dimensional parameter ${\bar{\text{V}}}_{\text{CV}}/{\bar{\text{V}}}_{\text{PCV}}$. Asterisk (*) denotes the significant protective effect of L-arginine pretreatment against Hcy-induced PMD. n = 7–13 per group; ** p<0.01 and *** p<0.001 (one-way ANOVA with Newman-Keuls's post-test). L-arg indicates L-arginine. Resulting values are expressed as mean ± SD (standard deviation).

The compromised endothelial NO-synthesis was previously suggested as the vasotoxic mechanism of elevated Hcy [15]. In addition, administration with an L-arginine was reported to attenuate this effect by promoting endothelial NO synthesis [16]. Therefore, we investigate the attenuating effect of L-arginine treatment against the Hcy-induced PMD using a parameter ${\bar{\text{V}}}_{\text{CV}}/{\bar{\text{V}}}_{\text{PCV}}$. As shown in Figure 4, in the absence of L-arginine pretreatment, ${\bar{\text{V}}}_{\text{CV}}/{\bar{\text{V}}}_{\text{PCV}}$ is dramatically decreased from 0.582 to 0.378 upon exposure to100 μM Hcy (p<0.001). This effect is fully blocked by pretreatment with 50 μM L-arginine, which gives rise to a ${\bar{\text{V}}}_{\text{CV}}/{\bar{\text{V}}}_{\text{PCV}}$ value of 0.585 (p<0.001) (Figure 4). Pretreatment with 10 μM L-arginine, on the other hand, results in an insignificant protective effect with a ${\bar{\text{V}}}_{\text{CV}}/{\bar{\text{V}}}_{\text{PCV}}$ value of 0.412 (p>0.05). These results suggest that pretreatment with 50 μM L-arginine protects against the onset of 100 μM Hcy-induced PMD, which is consistent with the previously reported protective effect of L-arginine against Hcy-induced vasotoxicity [16].

### Reduction in peripheral neutrophil adherence is associated with the protective effect of L-arginine pretreatment against Hcy-induced PMD

**Figure 5:**
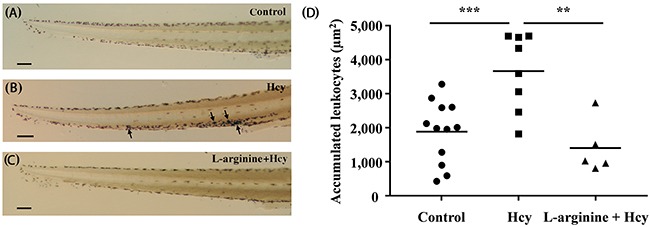
Accumulation of neutrophils in the tail region The stained neutrophils in the tail region of **(A)** control, **(B)** the 100 μM Hcy-exposed, and **(C)** the 50 μM L-arginine pretreated 100 μM Hcy-exposed zebrafish are visualized. Arrows indicate significantly aggregated neutrophils. **(D)** The areas with accumulated neutrophils in the tail region are quantitatively compared. n = 5–12 per group; **p<0.01 and *** p<0.001 (one-way ANOVA with Newman-Keuls's post-test). Control, control zebrafish without any treatment; Hcy, Hcy-exposed zebrafish; L-arginine + Hcy, L-arginine pretreated Hcy-exposed zebrafish. Scale bar denotes 100 μm.

Hcy-induced vasotoxicity results in the increased activation of neutrophils and subsequent adherence to vascular endothelium [2]. The increased adherence of neutrophils to microvessels contributes to Hcy-induced PMD through the volume-exclusion effect in Hcy-exposed lumen [17]. Therefore, Hcy-induced neutrophil accumulation is examined by selective staining of neutrophils with Sudan Black B. Sudan Black B is a lipid staining agent that stains neutrophilic granules to positively identify neutrophils [18]. Application of this dye to post-mortem specimens of the Hcy-exposed zebrafish reveals a higher number of stained neutrophils in and around the CV of their tail regions (black arrows), compared to those in the control zebrafish model (Figures 5A, 5B and 5D). A large majority of the neutrophils migrate into the CV, indicating their inflammatory activation. Given simplicity of the peripheral vascular structure of zebrafish tail region, only a small number of neutrophils adhered to the CV is required for development of PMD. Therefore, the Hcy-induced adherence of neutrophils is a contributing factor to Hcy-induced PMD. In support of this projection, the pretreatment with 50 μM L-arginine greatly reduces the accumulation of neutrophils in the CV region (Figures 5C and 5D). This result is consistent with the preventive effect of L-arginine pretreatment against the Hcy-induced PMD (Figure 4).

## DISCUSSION

The proposed dimensionless parameter ${\bar{\text{V}}}_{\text{CV}}/{\bar{\text{V}}}_{\text{PCV}}$ effectively characterizes Hcy-induced PMD in zebrafish. Peripheral microcirculation perfusion is selectively impaired relative to the systemic perfusion and the average cardiac output (Figures 2A and 2B). The dysfunction is also closely associated with the elevated peripheral accumulation of neutrophils (Figures 5B and 5D). This selective reduction in peripheral microcirculation is compensated by relative increase of the blood flow migrating from the SIV into the PCV (Figure 3). Furthermore, the pretreatment with 50 μM L-arginine significantly attenuates the onset of PMD (Figure 4) and the subsequent accumulation of neutrophils in the peripheral CV (Figures 5C and 5D). To the best of our knowledge, this is the first quantitative hemodynamic study on pathological effects of Hcy on the microcirculation of an intact *in vivo* disease model.

The degree of Hcy-induced PMD is assessed using four dimensionless parameters with varying Hcy level. Among the four parameters, ${\bar{\text{V}}}_{\text{CV}}/{\bar{\text{V}}}_{\text{PCV}}$ represents the function of peripheral microcirculation perfusion conveniently and effectively (Figure 2A). While ${\bar{\text{V}}}_{\text{CV}}/{\bar{\text{V}}}_{\text{ADA}}$ also effectively indicates the Hcy-induced PMD at higher Hcy doses (Figure 2B), ${\bar{\text{V}}}_{\text{CV}}/{\bar{\text{V}}}_{\text{PCV}}$ better reflects the development of PMD for all doses of Hcy in this study. Furthermore, ${\bar{\text{V}}}_{\text{CV}}/{\bar{\text{V}}}_{\text{PCV}}$ could be conveniently measured using only one ROI, while the evaluation of ${\bar{\text{V}}}_{\text{ADA}}$ requires additional experimental techniques due to high-speed flow velocity and violent pulsatility at DA. On the other hand, ${\bar{\text{V}}}_{\text{PCV}}/{\bar{\text{V}}}_{\text{PDA}}$ values are not varied under the Hcy-exposure conditions, leading to decreased ${\bar{\text{V}}}_{\text{PCV}}$, while ${\bar{\text{V}}}_{\text{PCV}}/{\bar{\text{V}}}_{\text{PDA}}$ is decreased under the same condition. This result indicates that Hcy-exposure reduces the pulsatility of zebrafish heart with relatively less effect on the average cardiac output. This implies that the end diastolic velocity (${\hat{\text{V}}}_{\text{EDA}}$) in the DA would increase in response to Hcy exposure. These results suggest that high peripheral vascular resistance would decrease ${\hat{\text{V}}}_{\text{PDA}}$ while increasing the end-diastolic volume and its pressure to pump out, because most RBCs would circulate around the proximal vessels to the heart. Similarly, decrease of ${\hat{\text{V}}}_{\text{PDA}}$ and increase of ${\hat{\text{V}}}_{\text{EDA}}$ in the intracranial artery were reported in the early stage of fetal circulation [19].

The present results show that the accumulation of neutrophils predominantly occurs in and around the peripheral CV (Figure 5B). The CV has been known to be prone to leukocyte adherence, because it is characterized in the regions of low wall shear stress (WSS) [20]. Immunological behaviors of activated neutrophils, including rolling and adherence, are also frequently observed in the region with low WSS [21]. Acute exposure to Hcy activates the expression of adhesion molecules, such as P-selectin, ICAM-1, L-selectin, and CD11b/CD18 [6, 7], which would cause exclusive accumulation of neutrophils in the region having those effects (Figure 5B). Furthermore, the adherence of neutrophils in peripheral vascular lumen accelerates peripheral resistance by pseudopod formation that plugs capillaries and narrows lumens size by volume exclusion effect [17]. In addition, the increased interactions between the activated neutrophils and RBCs increase local blood viscosity in the region, further increasing flow resistance [22]. In these regards, the present observation of the Hcy-induced peripheral neutrophil accumulation in the CV explains the onset of PMD (Figures 5B and 5D), as well as the protective effect of L-arginine treatment (Figures 4, 5C and 5D).

Another interesting observation in this study is the compensatory increase of blood flow in the SIV relative to the systemic perfusion upon the onset of Hcy-induced PMD. The reduced peripheral perfusion makes it possible to decrease the systemic perfusion based on the conservation of mass. However, systemic perfusion in the PCV is relatively preserved in a support of blood flow from the SIV (Figure 3), resulting in decrease of the ${\bar{\text{V}}}_{\text{CV}}/{\bar{\text{V}}}_{\text{PCV}}$ under PMD (Figure 2A). Isogai et al. (2001) examined vascular development in zebrafish model [23], and reported that its heart provides blood to the digestive organ via the supraintestinal artery and the SIV. This unique vascular network allows for direct circulation of blood flow from the heart to the PCV via the SIV, and hence makes the systemic perfusion preserved against PMD as long as the cardiac output is maintained.

In this study, we demonstrated unique advantages of zebrafish model in quantitative analysis of microvascular hemodynamic features, compared to conventional mammalian animal models. In addition to its ease of drug treatment and pathological assessment from its optical transparency, it also can be used as a reproducible platform for quantitative analysis of PMD. Specifically, while the vascular structures around the CV and PCV are highly consistent [12], the odd identification of the reproducible microvascular geometry with a consistent vasomotor response is nearly impossible in higher mammalian animal models [24]. In addition, since both ${\bar{\text{V}}}_{\text{CV}}$ and ${\bar{\text{V}}}_{\text{PCV}}$ can be non-invasively measured simultaneously in the same frame, zebrafish model has observational convenience for quantitative analysis of PMD. These advantages support that the present zebrafish model can be used as a potential high-throughput platform for drug screening against microvascular diseases.

## MATERIALS AND METHODS

### Sample preparation

Zebrafish were provided by Korea Zebrafish Organogenesis Mutant Bank (Daegu, Korea). The specimens were raised in freshwater containing 0.1 mM phenylthiourea to inhibit the melanization of zebrafish embryos. Cultivation conditions were as follows: room temperature of 28.0±0.5 °C and 14 h:10 h light-dark cycle. The fish were fed thrice a day after five dpf. Experiments were performed with 8–10 dpf zebrafish. All experimental procedures were approved by the Animal Care and Ethics Committee of POSTECH and were performed in accordance with the approved guidelines.

Hcy-exposed zebrafish samples were prepared by administering 10, 50, 100, 200, or 500 μM D, L-homocysteine solution (Sigma Aldrich, USA) to 9-dpf zebrafish for one day. To investigate the effect of NO against Hcy-induced PMD prior to Hcy treatment, L-arginine-pretreated zebrafish samples were prepared by administering 10 or 50 μM L-arginine solution (Sigma Aldrich, USA) to 8-dpf zebrafish for one day. A similar procedure was followed after L-arginine treatment.

### Experimental setup

A 10-dpf zebrafish was anesthetized by short exposure to 0.02% tricaine and mounted on 5% methylcellulose. Velocity field information and the number of RBCs in vessels were measured with sequential images, which were captured by an inverted microscope (Zeiss Axiovert 200, Zeiss, Germany) with a 20× (NA = 0.4) objective lens. The test vessel was illuminated with a halogen lamp. Sequential images were acquired in the center plane of the vessel by a high-speed CCD camera (Photron Ultima APX, Photron, Japan) at a frame rate of 125 fps. Image processing was performed with Image J software. All experiments were performed at room temperature (28 °C).

### Micro-PIV measurement

The velocity of RBCs as trace particles was measured by a micro-PIV technique. Unlike conventional PIV techniques that utilize a laser, this study adopted volumetric illumination with a halogen lamp in a consideration of low-depth of focus of the microscope lens. Velocity field information was obtained by a FFT-based cross-correlation PIV algorithm with a multigrid interrogation window of 64 ×16 pixels and 50% overlap. This technique can be applied to blood flows at high hematocrit levels to extract velocity vectors because each interrogation window should have at least several RBCs as tracer particles. Micro-PIV analysis was conducted with commercially available PIVview software (PIVview 2C, PIVTEC, Germany).

### Digital motion analysis

Sequential images are composed of static and dynamic pixels. Gray scale values of static pixels remain constant in consecutive images, whereas those of dynamic pixels are altered by moving obstacles. Subtracting static pixels yields a gray scale value of zero, whereas subtracting dynamic pixels yields gray scale values of 1 to 255. The number of subtracted dynamic pixels caused by moving RBCs is used to visualize the spatial distribution of RBCs without background image and to measure the number of RBCs passing through the vessels. The detailed methodology of motion analysis is described in [14]. In contrast to the micro-PIV technique which requires flow images from blood with high hematocrit levels to obtain velocity field information, this motion analysis technique can analyze the flow characteristics of blood with both low and high hematocrit levels. The flow characteristics in the SIV at low hematocrit levels and in the PCV at high hematocrit levels were investigated using by this technique. The number of RBCs passing through the vessel was estimated at the center region of the vessel for 10 s. Digital image processing was conducted with MATLAB software (Mathworks, USA).

### Staining of neutrophils with Sudan Black B

Briefly, 10 dpf zebrafish were fixed with 10% formalin overnight at 4 °C. The fish were washed thrice with PBS solution for 5 min and incubated in Sudan Black B solution (Sigma Aldrich, USA) following a previously reported procedure [18]. The neutrophils stained with a non-fluorescent dye were not clearly distinguished from unstained pigments around the vessels, which makes it difficult to use an automatic digital image processing technique. Thus, Image J software was employed to manually evaluate the accumulation areas of stained neutrophils in the tail region behind the sphincter.

### Statistical analysis

Statistical analysis was conducted with GraphPad Prism 7 software (Graphpad Software Inc., USA). Statistical significance was analyzed using one-way ANOVA with Newman-Keuls's post-test for multiple comparisons. Here, * indicates p<0.05, ** p<0.01 and *** p<0.001.
